# Supplementary figures and images for: Quantitative and functional interrogation of parent-of-origin allelic expression biases in the brain
Source: eLife. 2015 Jul 3;4:e07860. doi: 10.7554/eLife.07860 (PMC4512258; doi:10.7554/eLife.07860)

ENSMUST00000024599.7

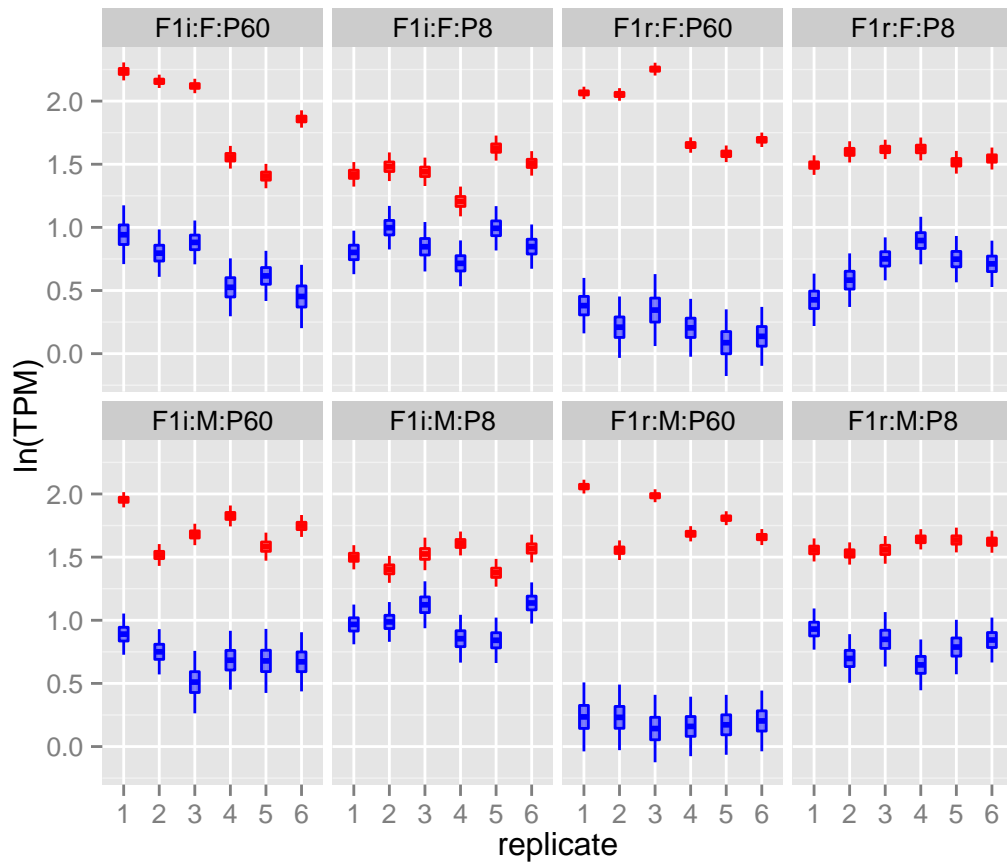

Supplement: Source code 1. — The compressed source code folder, braimSourceCode.zip, includes the BRAIM R code along with example parameters and output and a help readme file which explains how to run the code. DOI: http://dx.doi.org/10.7554/eLife.07860.020 [file elife07860s002.zip › braimSourceCode/ENSMUST00000024599.7.pdf]

ENSMUST00000125366.1

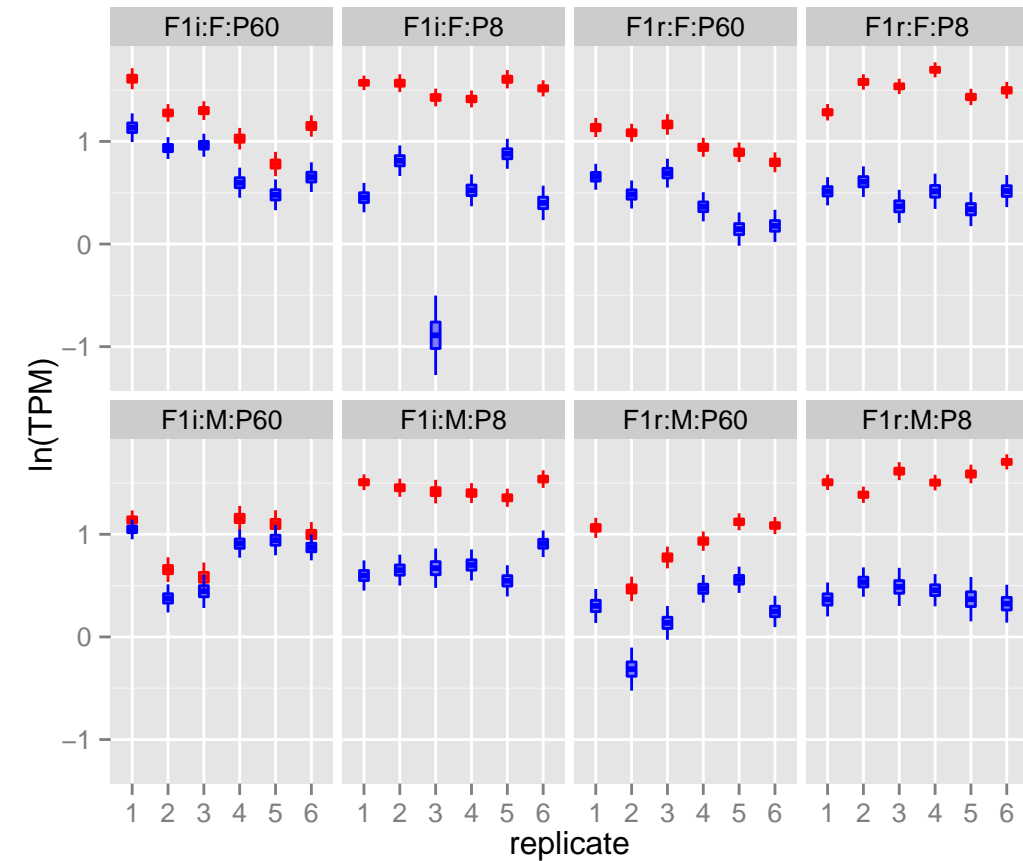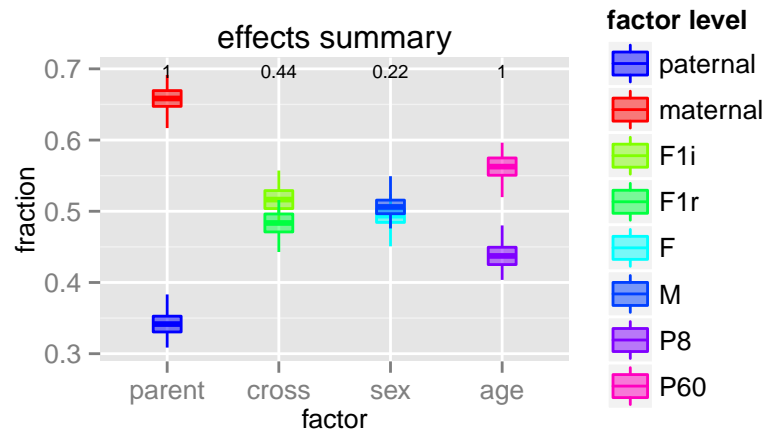

Supplement: Source code 1. — The compressed source code folder, braimSourceCode.zip, includes the BRAIM R code along with example parameters and output and a help readme file which explains how to run the code. DOI: http://dx.doi.org/10.7554/eLife.07860.020 [file elife07860s002.zip › braimSourceCode/ENSMUST00000125366.1.pdf]
